# Health-Related Quality of Life and Quality of Sexual Life in Obese Subjects

**DOI:** 10.1155/2014/847871

**Published:** 2014-02-23

**Authors:** Eleonora Poggiogalle, Luca Di Lazzaro, Alessandro Pinto, Silvia Migliaccio, Andrea Lenzi, Lorenzo M. Donini

**Affiliations:** ^1^Medical Pathophysiology, Food Science and Endocrinology Section, Department of Experimental Medicine, “Sapienza” University of Rome, Piazzale Aldo Moro 5, 00185 Rome, Italy; ^2^Department of Movement, Human and Health Sciences, University of Rome “Foro Italico”, 00195 Rome, Italy

## Abstract

The increased prevalence of obesity represents, currently, one of the major public health issues, due to its consequences on physical and psychological health status as well as on the psychosocial functioning. As defined by the World Health Organization, sexual health is “a state of physical, emotional, mental, and social well-being in relation to sexuality.” The aim of the present study was to explore the relationship between sexual life in obese subjects and quality of life, psychological status, and disability. *Methods*. 95 obese subjects were recruited from June 2012 to February 2013 and underwent physical examination and measures for the assessment of quality of life, sexual life, psychological status, and disability. *Results*. In obese subjects sexual life was related to gender, age, psychological status, disability, and quality of life. *Conclusion*. As obesity is a multifactorial disease, and is accompanied by multiple comorbidities, it is difficult to identify a single causative factor responsible for the impairment of sexual life in obese subjects; thus, a thorough, multidimensional evaluation including sexual function assessment should be performed in obese people.

## 1. Introduction

The increased prevalence of obesity represents, currently, one of the major public health issues, due to its consequences on physical and psychological health status as well as on psychosocial functioning [1–3]. On one hand, chronic diseases associated with excess fat are well known and documented [1, 2]; on the other hand, evidence is relatively scarce with respect to quality of life—including sexual life—in obese subjects [4, 5].

As defined by the World Health Organization, sexual health is “a state of physical, emotional, mental, and social well-being in relation to sexuality” [6]. Sexual dysfunction encompasses clinical syndromes that impair sexual functioning such as sexual aversion, dysfunctional sexual arousal and vaginismus in females, and erectile dysfunction and premature ejaculation in males [7].

Studies exist reporting that obese men and women tend to have greater problems in their sexual life when compared to their lean counterparts [8, 9]. A number of obesity-related comorbidities, such as dyslipidemia, hypertension, type 2 diabetes, and depression, are associated with sexual dysfunction [9–12]; hence, it is difficult to identify the role of obesity *per se *in the development of sexual dysfunction. Potential mechanisms explaining the association between obesity and sexual dysfunction include: endothelial dysfunction, metabolic syndrome and diabetes, endocrine disorders, obstructive sleep apnea syndrome, physical disability, and social and psychosocial problems [5].

Overweight and obesity have been identified as risk factors for sexual dysfunction only in men [4, 13, 14], whereas the relationship between female sexual function and excess fat remains to be better clarified [15–19].

Few studies described the impairment of quality of life in obese subjects [20]; sexual functioning is a determinant of quality of life, and excess fat could play a pivotal role also in the quality of sexual life [21, 22]. The aim of the present study was to explore the relationship between sexual life in obese subjects and quality of life, psychological status, and disability.

## 2. Materials and Methods

### 2.1. Study Population

Participants were recruited from June 2012 to February 2013 among outpatients referring to the High Specialization Centre for the Care of Obesity (named “CASCO”) at the Department of Experimental Medicine—Pathophysiology, Food Science and Endocrinology Section, Policlinico Umberto I—Sapienza University of Rome, Italy. Inclusion criteria were BMI ≥30 Kg/m^2^ and age ranging from 15 to 69 years. Oral and written informed consent was obtained from all the participants. The study was approved by the local ethics committee.

### 2.2. Demographics and Clinical Status

Medical history, with particular attention to obesity-related complications (insulin resistance, type 2 diabetes, hypertension, dyslipidemia, thyroid dysfunction, cardiovascular, osteoarticular, respiratory, gastrointestinal, and psychiatric diseases) and medication use, as well as demographic, social, and cultural information (age, gender, job, and education level), and smoking habits were obtained.

### 2.3. Anthropometric Measurements

All the subjects underwent physical examination. Anthropometric measurements were gathered following the procedures described in the Standard Manual for Anthropometric Measures [23]. Body weight was measured using a SECA scale (Hamburg, Germany) to the nearest 0.1 kg; height was measured using a SECA stadiometer (Hamburg, Germany) to the nearest 0.5 cm. Body mass index (BMI = body weight/height squared) was calculated. Skinfold thicknesses were measured using a Harpenden Skinfold Caliper (British Indicators Ltd, St Albans, Herts, UK), to the nearest 0.2 mm. Body composition fat mass (FM) and fat-free mass (FFM) were estimated by bioelectrical impedance analysis (BIA) using a single-frequency 50 kHz analyzer STA—BIA (Akern Bioresearch SRL, Pontassieve, FL, Italy). Measurements were performed following standardized procedures [24]. Estimation of FM and FFM by BIA was obtained using gender-specific BIA prediction equations developed by Sun et al. [25].

### 2.4. Measures for the Assessment of Quality of Life, Sexual Life, Psychological Status, and Disability

All the participants underwent the administration of four questionnaires.

(i) The Laval questionnaire—Italian version [26] consists of 44 items distributed in 6 domains: symptoms, activity/mobility, personal hygiene/clothing, emotions, social interaction, and sexual life (items n. 12 and 37). Each domain is scored on a 7-point Likert scale, higher scores corresponding to a better quality of life.

(ii) The O.R.Well-97 questionnaire [27] is composed of 18 items; for each item the patient is asked to score on a 4-point Likert scale the occurrence and/or severity of the symptom (occurrence) and the subjective relevance of the symptom-related impairment in one's own life (relevance). The items are related to three different areas: symptoms, discomfort, and impact of obesity on familial relationship, role functioning, and social network. We selected the items n. 2, 10, and 11 regarding sexual life.

(iii) The SCL-90 (Symptom Checklist-90) [28] is a scale including 90 questions exploring the presence and the severity of psychological symptoms occurred in the last week. Dimensions explored are somatization, obsessive-compulsive, interpersonal sensitivity, depression, anxiety, hostility, phobic anxiety, paranoid ideation, and psychoticism. Each answer is scored on a 5-point Likert scale. For the evaluation of sexual life, we selected the items n. 5, 21, 69, 84.

(iv) The TSD-OC (SIO obesity-related disability) test [29] is a questionnaire developed by the Italian Society of Obesity (SIO); it is made of 36 items divided into 7 sections: pain, rigidity, activities of daily living, housework, outdoor activities, occupational activities, and social life. Each question is answered using a visual analogue scale, with a score ranging from 0 (absence of difficulty) to 10 (the highest degree of disability) for each item.

### 2.5. Statistics

After the verification of the normal distribution of the variables, *t*-test was performed to describe differences between means of the groups. A linear regression analysis (Pearson's *r*) was performed to verify the association among continuous variables. Differences were considered to be statistically significant for *P* < 0.05. Statistical analysis was performed using SPSS 10.0 statistical software (SPSS Inc. Wacker Drive, Chicago, IL, USA).

## 3. Results

95 subjects (25 men and 70 women) were enrolled. Participants' demographic and clinical characteristics are summarized in Tables 1 and 2.

### 3.1. Sexual Life and Demographics

Men reported better scores than women in the domain “Sexual life” of the Italian version of the Laval questionnaire (*P* < 0.05). Analogous differences were reported in the scores obtained at the items n. 5; 21; 69; 84 of the SCL-90 questionnaire (*P* < 0.05).

A statistically significant relationship was found between age and scores at the selected items (n. 5; 21; 69; 84) of the SCL-90 scale addressed to sexual life (*r* = 0.24, *P* < 0.05); no significant differences were observed between genders and related to smoking habits.

### 3.2. Sexual Life and Anthropometric Parameters

Statistically significant correlations emerged between waist circumference (*r* = 0.33, *P* < 0.05) and the scores obtained for items n. 12 and 37 in the “Sexual life” domain of the Laval questionnaire—Italian version. The relationship between BMI and scores was obtained at the specific items about sexual life (n. 2; 10; 11) of the O.R.Well-97 questionnaire (*r* = 0.24, *P* < 0.05).

### 3.3. Sexual Life and Clinical Status (Table 3)

Subjects suffering from cardiovascular diseases had a worse sexual life (*P* < 0.05). No significant association was observed between sexual life and the remaining diseases or medication use.

### 3.4. Sexual Life and Quality of Life

In obese subjects a statistically significant relationship was shown between scores corresponding to the items n. 12 and 37 of the “Sexual life” domain, the total score of the Laval questionnaire—Italian version (*r* = 0.65; *P* < 0.01), and the other single domains of the same questionnaire: “activity-mobility”, “Symptoms”, and “Emotions” (*P* < 0.01), as well as total score (*r* = −0.61, *P* < 0.01) and scores of “Occurrence” (*P* < 0.01) and “Relevance” (*P* < 0.01) at the O.R.Well-97 questionnaire (*r* = −0.61, *P* < 0.01).

### 3.5. Sexual Life and Psychic Discomfort

Scores of the “Sexual life” domain of the Laval questionnaire—Italian version were statistically significantly correlated with the total score at the SCL-90 questionnaire and its single domains: “Somatization”, “Obsessive-compulsive”, “Interpersonal sensitivity”, “Depression”, “Anxiety”, “Hostility”, “Phobic anxiety”, “Paranoid ideation”, and “Psychoticism” (all: *P* < 0.01).

### 3.6. Disability

Scores regarding the “Sexual life” domain of the Laval questionnaire—Italian version were statistically significantly correlated with the total score at the TSD-OC test (*r* = −0.64, *P* < 0.01) and scores at its single domains: “Pain”, “Rigidity”, “Activities of daily living”, “Housework”, “Outdoor activities”, “Occupational activities”, and “Social life” (all: *P* < 0.01).

## 4. Discussion

In the present study, in obese subjects sexual life was related to gender, age, psychological status, disability, and quality of life.

We observed that sexual life in obese men was better than obese women; moreover, sexual life was influenced by age in our study population. These findings are in agreement with data provided by Laumann et al. [30], showing a greater prevalence of sexual dysfunctions in women (43%) when compared to men (31%); in addition, authors found a positive relationship between age and erectile dysfunction and lack of sexual desire in men, whereas women reported a reduction of sexual disturbances with ageing. From data analysis, in our study population, no differences related to gender emerged with respect to age and sexual life, but this finding may be due to differences in the male and female sample size (men were only 25).

In our study, we showed an inverse relationship between sexual life and BMI, and sexual life and waist circumference. With respect to men, our results are consistent with a number of previous studies [13, 18, 21, 31], demonstrating the association between obesity and erectile dysfunction, with obvious consequences in the global sexuality. In accordance with data in our study, Morotti et al. [32] observed an impairment in sexual function in overweight and obese women when compared with their lean counterparts. In a paper by [33], even though a negative relationship was found between body weight and sexual function, no association appeared between central fat distribution and sexual dysfunction. In contrast, in our study, waist circumference negatively correlated with sexual function, and it seems to be in line with the association between increased BMI and reduced sexual health, both showing a relationship between adiposity indices and sexual dysfunction.

In line with our results, cardiovascular disease was demonstrated to influence sexual function in both males and females [34]. Neurovascular factors seem to have a causal role linking cardiovascular disease and sexual dysfunction in both genders [35], but the evaluation of these peculiar causative aspects was beyond the objective of our study.

A relevant finding in the present study was the strict relationship between sexual life and the global quality of life. This connection has been previously described in obese individuals [36, 37]. Moreover, independently of body weight, in a large sample of subjects [30], poor physical and emotional quality of life was associated to a higher prevalence of sexual dysfunction in both the genders, and, in a sort of vicious cycle, an impaired sexual function was connected to a diminished individual and social quality of life.

Another important observation in our study was the association of sexual life and mental health, consistently with extant literature [22, 38–40]. In addition, in a number of studies, the association between obesity and psychological distress has been described [41, 42]. In particular, in our sample of obese subjects, the obesity-related psychological distress, as well as other psychological characteristics in obese subjects, may account for the positive relationship between psychic discomfort and sexual dysfunction in our study.

An interesting aspect explored in our study was the relationship between sexual dysfunction and disability in obese individuals. Functional disability in osteoarticular and neurologic diseases is known to affect sexual function. In our study, we reported that obesity-related disability, assessed through a specific tool (the TSD-OC test), was related to sexual dysfunction.

The main limitation to our study was the evaluation of sexual life and dysfunction-assessed using single items from different tools, without exploring the underlying disturbances. However, the aim of our study was to investigate the presence of sexual discomfort related to obesity, independently of any other potential cause. Another limitation is represented by the composition of the study sample that was not homogeneous in terms of gender. Sexual dysfunction takes different forms in men and women but the small size, in particular of the male subgroup, may account for the impossibility to perform separate analysis to identify gender-related differences in correlations between sexual life and quality of life, psychological status, and disability. Finally, probably due to the small sample, we did not find any correlation between sexual life and smoking habits, medication use, or hypogonadism.

## 5. Conclusion

As obesity is a multifactorial disease and is accompanied by multiple comorbidities, it is difficult to identify a single causative factor responsible for the impairment of sexual life in obese subjects; thus, a thorough, multidimensional evaluation including sexual function assessment should be performed in obese people.

Obesity is a disabling disease with significant sequelae in multiple domains [43], and our results suggest the need of awareness toward obesity-related disability and its impact on sexual life in obese subjects.

## Figures and Tables

**Table 1:** Demographic and clinical characteristics.

	Total sample	Males	Females	*P*
Population Study (no.)	95	25	70	
Age (years)	44.2 ± 13.5	41.1 ± 13.9	45.3 ± 13.3	∗∗
BMI (Kg/m^2^)	41.2 ± 8.2	43.9 ± 11.0	40.3 ± 6.4	∗
Waist circumference (cm)	118.4 ± 14.3	131.4 ± 20.2	113.8 ± 12.1	∗∗
Fat mass—BIA (%)	43.9 ± 4.8	39.8 ± 6.0	45.4 ± 4.3	∗∗
Fat mass—anthropometry (%)	44.3 ± 6.2	40.2 ± 8.5	45.8 ± 5.8	∗
Obesity-related diseases				
Type 2 diabetes mellitus (no.)	10	4	6	
Cardiovascular disease (no.)	10	3	7	
Hypertension (no.)	31	8	23	
Osteoarticular disease (no.)	40	9	31	
Respiratory disease (no.)				
Total (no.)	28	8	20	
OSAS (no.)	19	6	13	
Hypogonadism (no.)	3	3		
Medications				
CPAP therapy (no.)	5	1	4	
ACE inhibitors (no.)	9	2	7	
ARB (no.)	13	4	9	

*P < 0.05; **P < 0.01.

BMI: body mass index; Fat mass—anthropometry: assessed by the measurement of skinfold thicknesses (Lohman et al. [23]); fat mass—BIA: assessed through bioimpedance electric analysis; OSAS: obstructive sleep apnea syndrome; ACE: angiotensin converting enzyme; ARB: angiotensin II receptor blockers; CPAP: continuous positive airway pressure.

**Table 2:** Correlations between sexual life domain (at Laval questionnaire, SCL-90 test, and O.R.WELL questionnaire) and quality of life, psychological status, and disability.

	Laval questionnaire	SCL-90 Test	O.R.Well questionnaire
	Items: 12-37	Items: 5-21-69-84	Items: 2-10-11
Age	−0.18	0.24*	−0.03
Body composition			
Waist circumference	0.33*	−0.21	0.02
Fat mass—anthropometry	−0.11	0.23	0.15
Fat mass—BIA	−0.12	0.17	0.18
BMI	−0.11	−0.03	0.24*
Laval questionnaire			
Total Score	0.65**	−0.62**	−0.51**
Activity-mobility	0.70**	−0.52**	−0.51**
Symptoms	0.55**	−0.51**	−0.37**
Emotions	0.68**	−0.67**	−0.48**
O.R.Well-97 questionnaire			
Total score	−0.61**	0.63**	0.77**
Incidence	−0.51**	0.56**	0.69**
Relevance	−0.62**	0.63**	0.76**
SCL-90 test			
Global score index	−0.65**	0.82**	0.49**
Somatization	−0.58**	0.57**	0.37**
Obsessive-compulsive	−0.60**	0.68**	0.49**
Interpersonal sensitivity	−0.59**	0.80**	0.50**
Depression	−0.68**	0.77**	0.51**
Anxiety	−0.61**	0.73**	0.41**
Phobic anxiety	−0.42**	0.68**	0.34**
Paranoid ideation	−0.49**	0.74**	0.38**
TSD-OC test			
Total score	−0.64**	0.49**	0.44**
Pain	−0.60**	0.49**	0.38**
Stiffness	0.62**	0.55**	0.42**
Activities of daily living	−0.30**	0.23*	0.19
Housework	−0.55**	0.43**	0.28**
Outdoor activities	−0.61**	0.41**	0.37**
Occupational activity	−0.50**	0.39**	0.37**
Social life	−0.51**	0.45**	0.48**

*P < 0.05; **P < 0.01

Legend: BMI: body mass index; TSD-OC test: Italian Society of Obesity test for obesity-related disabilities; fat mass—anthropometry: assessed by the measurement of skinfold thicknesses (Lohman et al. [23]); fat mass—BIA: assessed through bioimpedance analysis.

**Table 3:** Sexual life defined through Laval questionnaire and clinical status.

	Laval questionnaire			
	Total score	Score in sexual life domain	Item 12	Item 37
Type 2 diabetes				
+	4.4 ± 1	4.8 ± 2	4.4 ± 2	5.0 ± 2
−	4.5 ± 1	4.7 ± 2	4.0 ± 2	5.2 ± 2
Cardiovascular diseases				
+	4.0 ± 1	3.8 ± 1*	3.2 ± 2	4.4 ± 2
−	4.6 ± 1	4.8 ± 2	4.2 ± 2	5.3 ± 2
Hypertension				
+				
−				
Osteoarticular diseases				
+	4.4 ± 1	4.4 ± 2	3.9 ± 2	4.6 ± 2
−	4.6 ± 1	5.0 ± 1	4.2 ± 2	5.7 ± 2
Respiratory diseases				
+	4.3 ± 1	4.6 ± 2	4.0 ± 2	4.7 ± 2
−	4.6 ± 1	4.7 ± 2	4.1 ± 2	5.4 ± 2

*P < 0.05.
